# Adolescent attitudes to illicit drug use, education and support: a qualitative study in the United Kingdom

**DOI:** 10.3389/fpubh.2026.1951002

**Published:** 2026-10-06

**Authors:** Chris Retzler, Aqsa Naser, Rachel Byrne, Osahon Ogbeiwi, Zoe McCluskey, Rebecca Gunn, Michael Doyle

**Affiliations:** 1 Department of Social and Psychological Sciences, School of Human and Health Sciences, University of Huddersfield, Huddersfield, United Kingdom; 2 South West Yorkshire Partnership NHS Foundation Trust, Wakefield, United Kingdom; 3 Department of Community Medicine, University of Medical Sciences, Ondo, Nigeria; 4 Public Health, Kirklees Council, Huddersfield, United Kingdom

**Keywords:** attitudes, drugs, education, illicit substance, substance use, young people

## Abstract

**Background:**

This study aimed to explore young people's knowledge and attitudes around illicit drug use, its prevention, and the barriers and facilitators to accessing support, with the aim of informing the development of preventative interventions that resonate with young people and address their specific needs and concerns. By drawing on these insights, interventions can be tailored to be more engaging, relevant and impactful for young people.

**Methods:**

A qualitative approach was used to analyse data collected from focus groups and interviews with young people (*N* = 97) in the United Kingdom, living in areas of socioeconomic disadvantage. Questions focused on the knowledge of young people around substances, the sort of substance use prevention education, or interventions, they would see as beneficial, and the obstacles and facilitators to accessing support for issues around substance use.

**Findings:**

The findings suggest that prevention strategies which are likely to appeal to young people should provide non-stigmatizing, harm-reduction approaches to prevention education, which are provided as early as possible. Interventions should provide the skills to enable autonomous decision making, consider including parents/families in education and include trusted sources of support such as those with experience and knowledge of substance use.

**Conclusions:**

This study informs the development of preventative interventions for young people by providing insight around the knowledge and views of young people in the United Kingdom. It highlights the importance of taking a multifaceted approach to prevention that considers individual, social, and systemic factors.

## 1 Introduction

During adolescence, many young people engage in high-risk behaviors, and experimentation with illicit substances remains common (1). However, early substance use is associated with risky and criminal behaviors, poorer physical and mental health outcomes, drug-related fatalities, educational disengagement, offending behavior, and an elevated risk of chronic drug-related harm in adulthood (2–4). In addition, substance use is not uniformly distributed across populations with young people experiencing deprivation, adverse childhood experiences, poor mental health, family substance use, or social disadvantage facing substantially elevated risk of drug use and associated harms (5). Although there are some positive indications that prevalence among young people has recently declined in the UK, prevention remains an important priority and there remains little evidence regarding effective means of preventing early experimentation with illicit substances (6).

Drug prevention strategies in the UK have evolved considerably over recent decades. While earlier approaches often relied on abstinence-based or fear-arousal messaging, current policy advocates a whole-system approach that combines education, family support, community engagement and early intervention (1, 2). However, evidence suggests that providing information alone is unlikely to change behavior. Instead, programmes that develop social competence, decision-making, resilience and resistance to peer influence are more effective in reducing substance use (3). This paper therefore focuses on understanding young people's knowledge and attitudes around illicit drug use, the types of substance use education and early support, they believe would help them make informed decisions, and the barriers and facilitators to accessing trusted advice or support if they have concerns about substance use. This knowledge is important for developing preventative interventions that resonate with young people, support informed decision-making and reflect their specific needs and experiences (4, 5).

Previous sociological literature on drug “normalization” suggests that recreational drug use in Britain has become embedded in mainstream leisure and peer cultures, moving beyond marginalized subcultures (6). Young people are likely to be regularly exposed to substance use through the media, older siblings or even parents, and interventions must therefore be designed which are culturally sensitive, authentic, relatable, and informed by young people themselves. This raises the question of what young people think drug education should look like. A UK survey revealed that pupils want drug education to be honest and realistic, moving beyond simple anti-drug advice (7). Consistent findings from numerous studies emphasize the importance of this approach (8, 9) and suggest that young people are keen to learn about the physical, emotional, and broader life consequences of drug use (10). Valuable insight can also be provided by young people who are experts by experience as involving these individuals in the development of drug education programs is likely to enhance relevance and engagement with the materials for other young people (11, 12).

Over a 30-year period Wright and Pearl (13) collected data around the experience and knowledge of young people (aged 14–15) about illicit drug use in Wolverhampton, UK. Over the period of the study (1969–1999) they saw a rapid increase in experience of illicit drugs, but importantly young people's knowledge around the dangers of drug use remained limited. When asked about the reasons for taking drugs, young people emphasized the importance of social influences, to cope with stress, curiosity and relief of boredom. The study also highlighted that TV was the most frequently mentioned source of information about illicit drugs, followed by friends and talk in school. Wright and Pearl (13) concluded that effective education should be developed to combat the levels of use and ignorance of the dangers, amongst young people.

Accessing help around drug use can be an important step in reducing later harms, particularly as we know that attitudes to substance use and help seeking are often established early in life (14), but little is known about the factors young people identify as the barriers and facilitators to seeking help. Most research examining help-seeking has focused on young people with established substance use disorders (SUD), exploring the barriers and facilitators to accessing treatment or specialist services. For example, a recent systematic review found that young people with SUD often fail to recognize the need for treatment and typically only access services after encouragement from parents or other adults (5). Similarly, Berridge et al. (15) found that young people were more likely to seek help when they perceived services to be approachable, confidential and non-judgemental. However, comparatively little research has explored what young people perceive as supportive before substance use becomes problematic, including the types of education, trusted relationships and early sources of support that may help prevent harmful substance use. This distinction is important, as prevention seeks to equip young people with the knowledge, skills and confidence to make informed decisions before formal treatment or specialist support is required.

Despite considerable research examining the prevalence, risk factors and consequences of adolescent substance use, relatively little qualitative research has explored how young people themselves believe substance use education and support should be designed, particularly within UK populations and among those living in areas of socioeconomic disadvantage. Existing prevention programmes are frequently developed from professional or policy perspectives, yet there is increasing recognition that involving young people in intervention development can improve the relevance, acceptability and effectiveness of prevention efforts (4). Understanding young people's knowledge, attitudes and preferences is therefore essential for developing educational interventions that reflect their lived experiences and meet their needs.

The present study used a qualitative approach to better understand the knowledge and views of young people about illicit drugs. By drawing on these insights, interventions can be tailored to be more engaging, relevant and impactful for young people. We had three broad areas of interest: (1) What do young people know about drugs? (2) What sort of substance use prevention education or interventions do young people see as useful? (3) What factors do young people perceive as facilitating access to advice and support when they have concerns about substance use?

## 2 Materials and methods

### 2.1 Participants

Nine semi-structured group interviews were conducted with young people aged 12–20 (*n* = 88) in school and youth groups with the aim of gaining insight into their knowledge about illicit drugs, views on drug education and support for issues related to substance use. A summary of the focus groups is provided in Table 1. The school samples had an average of 22% (range: 5.6–45.2%) of pupils whose first language was not English and 33% (range: 24.8–46.1%) of pupils eligible for free school meals over the last 6 years. The existing groups were drawn from targeted youth services, which typically engage with young people from more disadvantaged backgrounds although no further data was collected on them.

**Table 1:** Focus group demographics.

Code	Type of group	*N* (school year/age)
SA	Secondary school	5 (year 10)
SB	Secondary school	12 (year 10)
SC	Secondary school	9 (year 8)
SD	Secondary school	14 (year 9)
SE	Secondary school	18 (year 9)
SF	Secondary school	19 (year 10)
D1	Secondary school SEND group	6 (aged 15 years)
D2	LGBTQ + youth group	13 (aged 14–20 years)
D3	Alternative provision	2 (14 and 15 years)

To gain the perspectives of young people with more experience of drug use, we also conducted individual semi-structured interviews (*n* = 9). These individuals were aged between 15 and 21 years and were recruited from a local drug and treatment service which supports young people in Kirklees, UK.

Full ethical approval was gained from the School Research Ethics and Integrity Committee at the University of Huddersfield. Interested students were sent letters of consent to be signed by their carers. The letters contained information about the study and consent to participate in the focus groups. In cases where a signed carer consent was unavailable such as those young people interviewed individually, young people were assessed using Gillick competency (16).

### 2.2 Data collection

Convenience sampling was used to recruit young people to participate in the focus group discussions from schools and community settings in the Kirklees district. We asked teachers in the schools to identify students that were diverse and representative of the wider population in relation to ethnicity, gender, and age. We attempted to minimize selection bias by explaining the purpose of the research to teachers so that they understood that we didn't want them to select their best pupils, as we wanted to gain a broader understanding of young people's views, not just those of pupils who are high achievers or most likely to interact with us. The focus groups were facilitated by group leads from a youth participation team at Kirklees Council, who already had relationships with young people in these settings. To allow young people to be able to express themselves fully, there were no teachers or group leaders present at these groups. The duration of each group was around 60 min and located in classrooms or community settings. Community settings included alternative provisions and LGBTQ+ groups with which our partners had existing relationships but also who represent groups who are at a heightened risk of substance use (17, 18).

The focus groups began with icebreaker activities, during which the young people could either engage in relevant topics or respond to questions ranging from personal preferences like their favorite bands to addressing significant issues faced by young people in today's society. These icebreakers served to engage the young people and encourage them to open up (19). Next, open-ended questions explored substance knowledge, young people's views on drug education and support around drug use (see Table 2 for questions). The questions were provided in one of two ways dependent on the size and makeup of the group. For larger groups, questions were written on scraps of paper and placed into a bag, from which each of the young people was then asked to draw a question and read it aloud before all members could contribute a response. In smaller group settings, practitioners adopted a conversational approach when posing questions to actively involve young people. The approach used was intended to create a comfortable environment and to encourage free expression.

**Table 2:** Focus group questions.

Question	Focus
What do young people know about drugs?	Baseline knowledge and awareness
Why do you think young people might use drugs?	Perceived motivations for use
When do drugs become a problem?	Perceptions of harm and thresholds
What would help people make clearer choices about drugs?	Decision-making supports
At what age should you learn what about drugs (what they look like, what they do, risks, law, decision making)?	Timing and content of drug education
Who is the best person to help you learn about drugs?	Preferred sources of education
Where might young people look for help around drugs?	Help-seeking pathways
What might stop young people asking for help around drug use?	Barriers to help-seeking

### 2.3 Analytic procedure

Qualitative data from the recordings of the individual interviews and focus groups were transcribed intelligent verbatim to preserve the meaning but remove redundant words and sounds such as pauses, stutters and repetitions and other irrelevant material. The responses were analyzed using thematic analysis which is a method to identify and present recurring patterns or themes in data (20). This included careful in-depth reading of the transcripts and the application of descriptive codes to relevant sections of the data. These codes were then categorized and labeled into broader themes and investigator triangulation was used to reduce individual bias. Next, the themes were reviewed to make sure they provided an accurate representation of the data and generalized into the final themes. Throughout the process, colleagues not involved in the analysis, provided peer debriefing around the themes and interpretations. Schools were labeled as SA–SF, groups recruited to ensure greater diversity were coded as D1–D3, and interviewees were assigned labels I1–I9 (Please see Table 1 for summary).

### 2.4 Reflexivity

Critical reflexivity is highly important in action research, particularly when working with young people (21, 22). The focus groups were facilitated by group leads from a youth participation service who already had working relationships with some of the groups. This meant that they had less research training than the core research team, for which we provided full briefing around the aims of the project, but they were very experienced in eliciting young people's views. The data from the focus groups, as well as interviews was collected and analyzed primarily by the research team. The research team was made up of highly educated people with backgrounds in research, Psychology and Public Health which is likely to have meant that they were considered outsiders by the young people. This may have affected rapport building and open discourse, although we attempted to mitigate this using open questions and activities which encouraged interaction between the researcher and young person. During the analysis, reflexive thematic analysis was supported throughout by regular team discussions, which created an intentional space for professional assumptions to be surfaced, examined, and revised in response to youth insight.

## 3 Findings

### 3.1 What do young people know about drugs?

As would be expected in a sample drawn from across adolescence, across both the interviews and focus groups, young people had differing levels of knowledge about drugs. Although they were often able to identify common street names of some drugs (e.g., “smart whip,” “balloons,” “coke,” “weed,” “ganja”), they were less sure around terms such as cocaine and ecstasy.

Cannabis dominated discussion and was often treated as familiar, accessible, and in some cases not seen as a “real” drug. Young people spoke about it in casual, everyday terms:

*Small amounts of weed won't do you any harm* (SD.1).

*I don't know if weed is good or bad for you at the moment* (SB. 11).

*I think weed is very popular, loads of people use it* (SA.2).

*Cannabis …, you can get any strain, from anywhere—your corner shop, you can get it from your friend's friend, any phone call* (SA.2).

Similarly, young people were aware of some of the psychological and physical effects of drugs, including changes to the user's emotions, behavior and the body. They suggested that these were some of the reasons why someone might use drugs, including positive effects on mood, to take their mind off things and to help them cope. The young people also commonly mentioned that drug use was a method of ‘escapism' which is why they thought many people used drugs.

*For pleasure! It's used in social situations with friends. It's fun to do with friends* (D2.21).

*To get their mind off stuff* (D3.21).

*Like you might smoke weed for anxiety* (I9).

*I've been told smoking weed makes people more creative so some people smoke it before exams* (SC114, aged 16 years).

*To forget about their problems* (SF.3).

The young people perceived drugs as harmful but could also be used medicinally or to enhance creativity. They also showed some understanding of the progression of drug use toward addiction.

*As time goes on, you'll do it more and you'll get addicted to it* (I2).

However, across the focus groups and interviews when the young people were asked about the effects of cocaine almost all young people said that they “didn't know” or that they “couldn't explain”. It was also suggested by the young people that they, and their peers, are often more aware of the short-term effects of drugs such as “getting high”, “paranoia” and “hallucinations” rather than the long-term effects and the risks that may come with drug use:

*People know more about how it makes them feel, rather than the negative side effects like long term effects on physical or mental health* (I6).

*They don't know as much as they think they do* (I4).

*I know what it does to you, but can't explain* (D3.13).

*Can get illnesses from smoking it but don't know what illnesses* (SA.13).

Young people, particularly those with experience of drugs, recognized the link between mental health and drug use, such as causing anxiety and depression.

*It makes you more depressed* (I4).

*It has negative effects like depression and anxiety, it can make you drift away from your family* (I1).

When asked about the reasons for drug use, young people suggested that many young people do it to earn peer respect and to keep up appearances, to show off or look cool, pleasure, curiosity, experimentation or to fit in with their friends. Most young people highlighted the pressure of “fitting in” or appearing cool' in front of their peers which could lead to them to use substances to “seek approval” from their friends. They also discussed the challenge of being socially influenced or pressured:

*Depends on the type of person they are, as to whether they can say no to friends* (SB.23).

*Peer pressure is also likely, especially if you're trying to look cool feeling you need to do drugs to fit in or to not get bullied, when other people are doing it* (D2.24).

Young people also suggested that they think others may use drugs to imitate others in their life such as friends and family.

*Might see parents or friends take it so they want to try* (SC.22).

### 3.2 What sort of substance use prevention education or interventions do young people see as useful?

Almost all of the data from interviews and focus groups found that young people thought that they should learn about drugs at “a young age.” The young people stated that most of them were exposed to drugs as soon as they started high school (i.e., from 11 years old). Based on this, they thought that drug education would be best just before entering high school and then during the first year.

*I think we should learn early because a lot of people start using them young* (SE.51).

*As soon as you hit high school (around 12 years old) you should be taught about drugs* (SA.51).

*When you start going out by yourself and coming to school yourself as that's when a lot of people are alone with other young people and would been more in danger of becoming influenced without having a parent there with them. You get a lot of vulnerable people that get targeted by themselves and on their way to school* (SD.59).

Interestingly there was awareness amongst some of the young people that exposure to drugs was likely to depend on the area in which they live:

*Depends on the area they live in. Some people are more exposed, so if you're living in an exposed area then you should learn from earlier. Some might not see it as commonly, so it might not be important for them to know about drugs* (SF.4).

When asked about what would help them make better choices around drugs, most young people agreed that they would like more education including the risks and effects. Young people who were no longer at school did not mention education as much but did highlight that they would have wanted more information on drugs before using them. Interestingly when probed on the type of approaches to education that might work, many responses focused on the effectiveness of real-life scenarios to highlight the effects and experiences of drug use amongst substance users.

### 3.3 What do young people see as the obstacles and facilitators to accessing support for issues with drugs?

When asked about support around drug use, young people wanted support in making positive decisions to reduce harm, rather than being told to abstain from drugs entirely. Young people also discussed the idea of being given the responsibility to make their own decisions.

*I think if you give them biased information then children will go against it so if someone says drugs are really bad don't do it, then children will want to do them more* (SD.63).

Young people wanted to get drug information from people they trust, which included family and friends but also professionals, particularly those with relevant experience:

*Non-judgemental adults! Experts and professionals* (D2.61).

*I'd want to ask someone who works with drugs* (D1.77).

Young people identified a number of barriers to seeking support around drugs included being afraid to ask for information or help due to fear of punishment or trouble with authorities, fear of how school or carers will react, and fear of being judged or rejected. Young people also described a lack of trust as creating a barrier to seeking help. Most of the young people did not trust teachers, carers, local authorities such as social workers and police or most adults in their life, as they were “scared to get in trouble”.

*I don't think most people would like tell their parents because they don't want to get in trouble* (I9).

*Loads of people do them and when someone says they're coming in to talk about drugs, especially if you're from an area of authority such as a school, police or council, because it feels like you're gonna get in trouble when we need less trouble and more discussions* (D2.84).

*You don't want to go to parents if you've took them cos the parents will get angry* (SD.71).

However, there was some disagreement on trusting teachers presumably based on their personal experiences:

*You've got to find the teacher that understands you and knows you and one that you can get along with* (I1).

Peer influence was also identified by young people as a barrier to accessing support. The young people stated that they may not want to access help due to pressure from peers or someone they may have got the drugs from as they may have “got pressured into not saying something.” This theme of pressure from peers to not access help or inform anyone was common and they labeled it as “being controlled” by “someone who was giving you the drugs” or “if they felt in danger.” Similarly, they didn't want to be seen as “cowards” by their peers and they feared being judged for wanting to seek support.

*Feeling like they're a coward cos they're looking for help you are in high school so you should be responsible and you should be able to handle it* (SD.81).

*They might think they are going to get judged* (SD.82).

Young people were unanimous in identifying carers as a barrier, often mentioning the carer's ignorance of drugs and their likely reaction if they found out that their children were using illicit drugs including judging, scolding and punishment. Interestingly, young people felt that carers had a lack of awareness of drugs, incomplete drug education, provided misinformation and negative information.

*I don't think most people would like tell their parents because they don't want to get in trouble* (I9).

*Telling you off for finding out when you might genuinely need help. Its like school* (SD.64).

*Punishment doesn't always make things better or make people learn. It can just annoy people and push them away from support more* (SD.42).

## 4 Discussion

This study explored the knowledge base of young people from a relatively deprived area of the UK around illicit substances, what education or interventions young people saw as useful and finally what would help them access support around drugs. As might be expected, young people showed variable knowledge of drugs which generally increased with age. They understood that the reasons for drug use are diverse and include recreation, mental health and peer influence. They felt that educational interventions should start early, include education about the health risks, be authentic, focus on social skills, work with parents to increase their knowledge and build trust with young people and ultimately aim to provide them with the ability to make autonomous informed decisions about drug use. These findings provide evidence that can inform intervention choice and development going forward.

Many young people recognized the short-term effects of substance use such as “getting high” or “paranoia” yet struggled to explain longer-term consequences. Indeed, young people stated that they were unaware of the health risks of substance use and stated that, had they received better education, their attitude toward use may be different and they would be less likely to use substances. This aligns with earlier research showing that young people's knowledge tends to focus on immediate, experiential outcomes rather than abstract health risks (13) and is particularly worrying given that a lack of knowledge around the risks of substance use is likely to increase the chances of engaging in drug use (23). It is interesting that young people felt they weren't aware of the health risks despite current PSHE curriculum guidelines including the long term risks and effects of substance use (24). This is likely due to the developing adolescent brains prioritization of immediate rewards and sensation seeking experiences over abstract future health risks (25, 26).

The findings showed that the reasons for drug use were diverse, ranging from social influence and psychological problems to recreational motives and lack of knowledge. Social influence, particularly peer influence, was emphasized, indicating the significance of addressing social dynamics in preventative efforts, aligning with previous findings (27–29). Moreover, young people highlighted the bidirectional relationship between substance use and mental health in line with evidence showing associations between mental health indices and higher use of substances (30). Evidence suggests a complex relationship, particularly around early use, in which stress can lead to changes in the neural circuitry responsible for decision making and emotion regulation, while early drug use can also impact the developing brain, and lead to increased risk of later uncontrolled use (31, 32). Given the links between substance use and mental health, alongside the increasing levels of mental illness, further research will be required to better understand this relationship and interventions will need to address both in tandem.

Young people highlighted their preference for early education on substance use, preferably during primary school, which aligns with research showing that initiation of drug use at earlier ages has been linked to increased risks of poor mental/physical health, poor social functioning and an increase in delinquency (9). However, it should be noted that adolescent attitudes to drugs develop and change through teenage years (6, 9), and programme quality, relevance, and reinforcement may be more important than simply starting young (3, 33). In addition, young people themselves often say that mistimed or moralistic education feels irrelevant (7, 8).

Young people said that they would be more open to support if there were better trusted sources of support available, including non-judgemental adults, experts by experience and friends; highlighting the importance of a supportive network in addressing substance use issues and aligning with previous findings (11, 12, 15). Autonomy in decision making was also important to the young people; they were keen to be able to access support but wanted it to be on their own terms and come from trusted individuals who have relevant experience and knowledge of the area. Interestingly, many of the young people said that they trusted each other and would turn to each other for support, highlighting the potential of peer-educator approaches. Although previous research has found that carer strategies such as monitoring the whereabouts of young people can reduce substance use (34) the young people in this study disagreed. They emphasized the importance, of having the freedom to make decisions around substance use, not to simply be told not to use substances, or punished for using them, which is consistent with previous findings (8, 9). Young people in our study often described parents as difficult to approach about drugs which is interesting given that recreational substance use has become increasingly normalized across generations, with many parents themselves likely to be drug-aware or have personal experience of use (6).

In summary, young people highlighted the following considerations for educational interventions to reduce substance use:

- Young people wanted educational interventions to be implemented early, although educational packages must strike a balance between informing young people about drugs but not exposing them to information too early.
- The desire for autonomy emerged repeatedly—young people wanted the tools and information to make their own decisions, not imposed abstinence, reinforcing the potential of harm reduction and participatory approaches.
- Regarding the content of educational interventions, many young people valued real-life stories and insights from those with lived experience, preferring balanced, honest discussions over prescriptive “don't do drugs” messaging.
- Young people wanted interventions to help build the skills to deal with peer influence.
- Interventions should seek to educate families about substance use and include information around how to initiate conversations with young people about drug use.

While no single intervention incorporates all of the characteristics identified by young people in this study, several evidence-based programmes align with many of their preferences. Systematic reviews have shown that school-based interventions are most effective when they move beyond providing information about drugs and instead develop social competence, life skills and strategies for resisting peer influence (3, 35). One of the most widely evaluated programmes is Unplugged, a universal school-based intervention that combines factual drug education with activities to develop communication, critical thinking, decision-making and peer resistance skills. The programme also includes parental involvement and has been shown to reduce cannabis use, episodes of drunkenness and daily smoking (36). Similarly, the Life Skills Training programme provides self-management skills, social skills and drug information, and has consistently demonstrated positive effects on reducing substance use (37). Young people in the present study also emphasized the importance of autonomy, honest discussions and practical support rather than abstinence-only messaging. These preferences are reflected in harm reduction approaches such as the School Health and Alcohol Harm Reduction Project (SHAHRP), which has been shown to reduce risky alcohol use by supporting informed decision-making, as well as providing education and skills training (38). Collectively, these interventions align with the preferences expressed by young people in this study, suggesting that effective substance use education should combine accurate information with social and emotional skill development, peer influence resistance, trusted sources of support and family involvement where appropriate. These principles also informed the co-production and implementation of the Reducing Illicit Substance Use Project (RISUP) educational resources, for which the current study provided an evidence base on which to develop interventions (39–41).

### 4.1 Limitations

There are several limitations that should be considered. First, although the study sought an in-depth understanding of the views of young people, this was limited to a small geographical area of Kirklees in Yorkshire, UK. This is an area of relatively high deprivation with some areas falling within the most deprived 10% in England (42) and a higher-than-average proportion of residents from minority ethnic backgrounds (26.4%) (43). As such, the findings may not be fully generalisable to youth populations in other parts of the UK, particularly those living in less deprived or more demographically homogeneous areas. It should however be noted that many of our findings did align with those of studies in different populations (e.g., findings around young people's knowledge about drugs, the reasons for use, desire for early education, and trusted sources of support) suggesting that there may be some commonality across populations in these areas. Second, much of the data collection took place in school settings which could have impacted the answers of young people who may have been influenced by social desirability bias, especially given the reported lack of trust in school-based staff and services, citing concerns about confidentiality and perceived judgement. These dynamics may have constrained open dialogue and thus influenced the depth or candor of responses. Similarly, the young people were, in some cases, teacher selected, which could have led to selection bias with only “good” pupils chosen who may be relatively drug naïve. To counter this, we asked teachers to select a range of students with a mixture of abilities, although of course we don't know whether they followed this request. Future studies should consider random sampling or a stratified approach. Finally, due to the time constraints of the current project we were unable to employ procedures such as member checking, audit trails, or coding consensus during the analysis which would have strengthened the reliability of the findings.

### 4.2 Conclusion

This study aimed to inform the development of preventative interventions for young people by providing insight around the knowledge and views of young people in the UK, living in areas of socioeconomic disadvantage. It highlights the links between mental health and substance use, and the importance of addressing both when developing interventions. The findings suggest that prevention strategies which are likely to appeal to young people should provide non-stigmatizing, harm-reduction approaches to prevention education, which are provided as early as possible. Interventions should provide the skills to enable autonomous decision making, consider including parents/families in education and include trusted sources of support such as those with experience and knowledge of substance use. Overall, the study highlights the importance of taking a multifaceted approach that considers individual, social, and systemic factors in shaping substance use interventions.

## Data Availability

The raw data supporting the conclusions of this article will be made available by the authors, without undue reservation.
